# Trypanosomatid infections in captive wild mammals and potential vectors at the Brasilia Zoo, Federal District, Brazil

**DOI:** 10.1002/vms3.216

**Published:** 2019-11-19

**Authors:** Filipe C. Reis, Thaís T. C. Minuzzi‐Souza, Mariana Neiva, Renata V. Timbó, Igor O. B. de Morais, Thiago M. de Lima, Mariana Hecht, Nadjar Nitz, Rodrigo Gurgel‐Gonçalves

**Affiliations:** ^1^ Laboratório de Parasitologia Médica e Biologia de Vetores Faculdade de Medicina Universidade de Brasília Brasília Distrito Federal Brazil; ^2^ Fundação Jardim Zoológico de Brasília Brasília Distrito Federal Brazil; ^3^ Laboratório Interdisciplinar de Biociências Faculdade de Medicina Universidade de Brasília Brasília Distrito Federal Brazil

**Keywords:** Conservation, *Leishmania*, Translocation, *Trypanosoma*, Zoonosis

## Abstract

**Background:**

Conservation projects in zoos may involve translocation of captive animals, which may lead to pathogen spread. Neotropical mammals are important hosts of *Trypanosoma cruzi* and *Leishmania* spp. the etiological agents of Chagas disease and Leishmaniasis respectively. Studies of trypanosomatid‐infected mammals and vectors (triatomines and sandflies) in zoos are important for the establishment of surveillance and control measures.

**Objectives:**

We investigated trypanosomatid infections in captive wild mammals, triatomines and sandflies at the Brasília Zoo.

**Methods:**

We collected triatomines during active bimonthly surveys, sampled sandflies using light‐traps and obtained blood samples from 74 mammals between 2016 and 2017. We used quantitative PCR to detect trypanosomatids in vectors and mammals.

**Results:**

We found a colony of 19 *Panstrongylus megistus *in the porcupine unit and detected *T. cruzi *infections in five bugs. We captured 17 sandflies of four species including *Nyssomyia whitmani *and *Lutzomyia longipalpis*, but no *Leishmania *infection was detected. qPCR detected 50 *T*. *cruzi‐*infected mammals belonging to 24 species and five groups of mammals (Carnivora, Cetartiodactyla, Perissodactyla, Pilosa and Primates); *Leishmania* DNA was detected in 23 mammals from 15 species, mainly carnivores. We detected trypanosomatid infections in 11 mammals born at the Brasília Zoo.

**Conclusions:**

Our results suggest vector‐borne transmission of *T. cruzi* among maned wolves; measures to reduce the risk of new infections should therefore be taken. We also report sandfly presence and *Leishmania*‐infected mammals at the Brasília Zoo. Translocation of wild mammals in and out of the Brasília Zoo should consider the risk of *T. cruzi* and *Leishmania* spread.

## Graphical abstract

Conservation projects in zoos require translocations of captive animals, which may spread pathogens. We aimed to verify the occurrence of vectors (triatomines and sandflies) and to investigate Trypanosoma cruzi and Leishmania infections in captive wild mammals in the Zoo Brasília. We collected triatomines and phlebotomines and we sampled blood from 74 mammals from which we confirmed T. cruzi and leishmania infections.

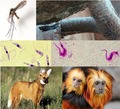

## 1 INTRODUCTION

Zoos play a key role in ex situ conservation by engaging visitors, community and employees (Barongi, Fisken, Parker, & Gusset, 2015). Scientific research and species reproduction are also fundamental activities in zoos. If necessary, reintroduction programs for captive animals are developed to increase the free‐living population and, in many cases, save species from extinction (Bowkett, 2009). Long‐term population viability often requires transfering animals among institutions for breeding (Barongi et al., 2015). However, the risk of pathogen transmission by moving infected individuals should be evaluated. The spread of zoonoses is among the potential consequences of translocation. Infectious diseases are transmitted between hosts by a variety of mechanisms, including direct, aerial and vector‐mediated transmission (Fèvre, Bronsvoort, Hamilton, & Cleaveland, 2006). Translocated animals can carry pathogens to the destination environment, acquire new ones during translocation or even become infected in this new environment (Leighton, 2002).

*Trypanosoma cruzi* infections are common in free‐living and captive primates of several species, with infection rates up to 83% (Bahia et al.., 2017; Lisboa, Dietz, Baker, Russel, & Jansen, 2000; Lisboa et al., 2006; Ziccardi & Lourenço‐de‐Oliveira, 1997). Triatomine colonization was reported in the Rio de Janeiro Primatology Center (Lisboa et al., 2004). Vector‐borne transmission of *T. cruzi* among captive Neotropical primates was recently described at the Brasília Zoo (Minuzzi‐Souza et al., 2016).

Phlebotomines were also reported in a Brazilian zoo, where high frequency of *Nyssomyia whitmani* was found (Teodoro et al., 1998). Moreover, Souza et al. (2010) reported the infection of six crab‐eating foxes (*Cerdocyon thous*) and a bush dog (*Spheotos venaticus*) by *Leishmania infantum* kept in the zoo of the Federal University of Mato Grosso. *Leishmania infantum* was also detected in *C*. *thous*, *Puma concolor* and *Panthera onca* at a zoo in the state of São Paulo (Da Silva Tenório et al.., 2011; Dahroug et al., 2010). After the death of a bush dog with Visceral Leishmaniasis at the Zoobotanical Foundation of Belo Horizonte, Minas Gerais State, 14 other canids kept in captivity at this institution were examined and four were positive for *L. infantum*, including one crab‐eating fox, one maned wolf (*Chrysocyon brachyurus*) and two hoary foxes (*Lycalopex vetulus*) (Luppi et al., 2008).

In the Federal District of Brazil (FD hereafter), estimates indicate high rates of triatomine infection by *T*. *cruzi* (Minuzzi‐Souza et al., 2018), which may be related to the enzootic transmission of this parasite in gallery forests. For example, 33% of 18 white‐eared opossums (*Didelphis albiventris*) were infected by *T*. *cruzi* in the forest adjacent to the Brasília Zoo (Gurgel‐Gonçalves et al., 2004). *Leishmania infantum*‐infected dogs have also been confirmed in FD (Cardoso et al., 2015), where sandflies are frequent in gallery forests (Ferreira, Minuzzi‐Souza, et al., 2015; Rapello et al., 2018) and houses (Carvalho, Bredt, Meneghin, & Oliveira, 2010). Studies of trypanosomatid‐infected mammals and vectors (triatomines and sandflies) in zoos are important for the establishment of surveillance and control measures for leishmaniasis and Chagas disease, including valuable information for animal health and management strategies such as translocations. Therefore, our aims were: 1) to identify triatomines and sandflies and their infection rates; 2) to determine the frequency of infection with *T. cruzi* and *Leishmania* spp. among captive wild mammals; and 3) to investigate vector‐borne transmission of these trypanosomatids at the Brasília Zoo.

## 2 MATERIAL AND METHODS

**Figure 1:**
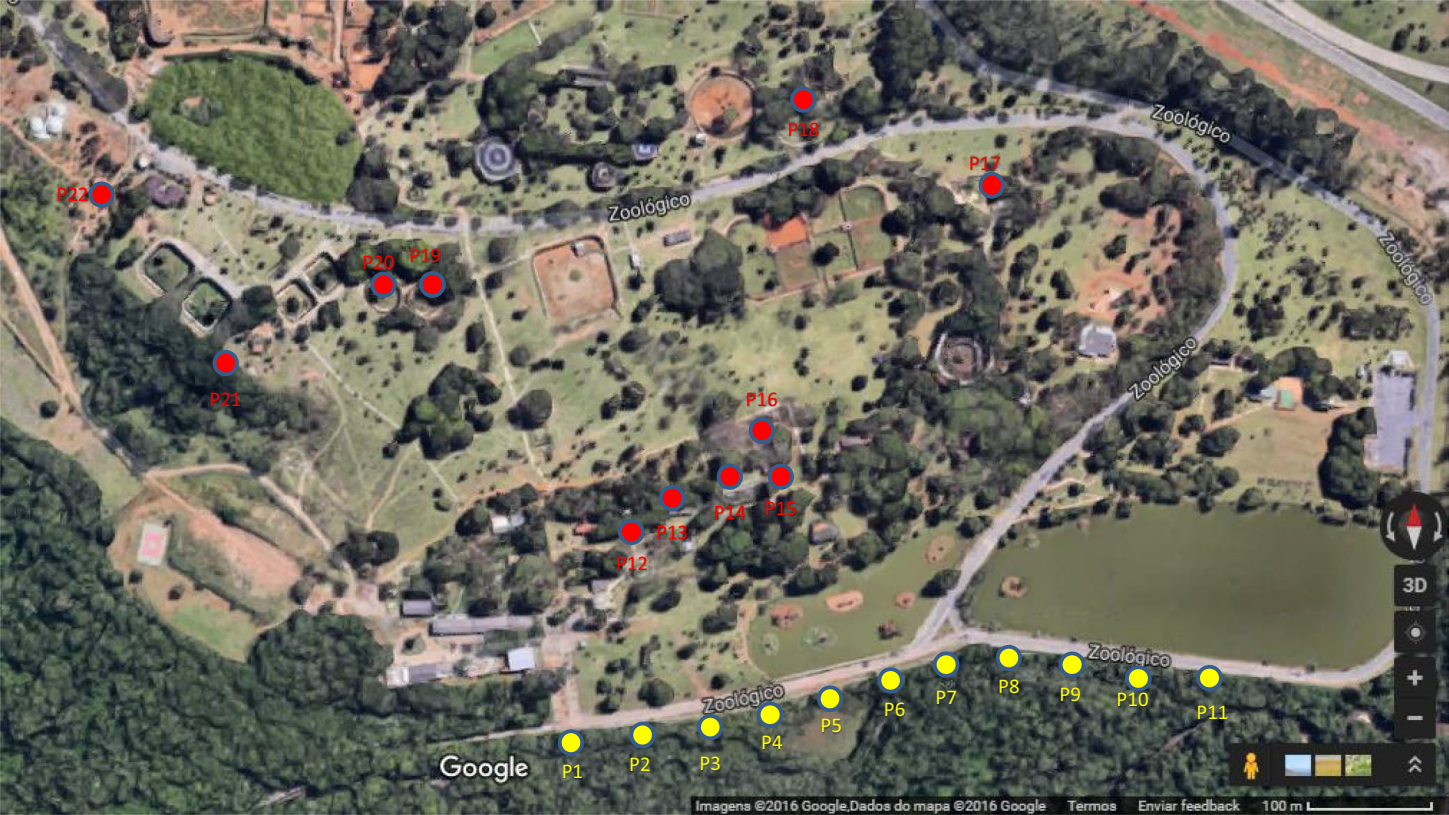
Satellite image of the Brasília Zoo area with sampling points for sandflies. The yellow dots are in the gallery forest and the red dots are located in the zoo's mammal units

The Brasília Zoo is in the Brazilian Federal District, Center‐West Region of Brazil (−15.8475°S, −47.9392°W). The institution was established on 1957 in an area of 619.7 hectares and has approximately 900 animals. We distributed HP light traps (Pugedo, Barata, França‐Silva, Silva, & Dias, 2005) in 22 points (Figure 1): 11 in the gallery forest and 11 in the zoo's units (primates, lowland tapir, crab‐eating fox, maned wolf, giant otter, giant anteater, puma, jaguar and Brazilian porcupine). At each point, we set one HP light trap daily at 5:00 p.m. and collected the trap on the following day at 7:00 a.m. for four consecutive days, bimonthly, between 2016 and 2017. In the last month, we used Shannon traps in all capture points where HP‐traps were previously installed. We separated sandflies by sex, date and location. We mounted whole males and female heads and distal abdomen segments following Forattini, Ferreira, Rocha Silva, and Rabello (1973); we then identified all specimens to species level using the keys by Galati (2017). We stored female thoraces and proximal abdominal segments in PBS 1× at −20°C for DNA extraction.

We sampled triatomines in mammal units bimonthly for four consecutive days, between September 2016 and September 2017. In addition, there was daily surveillance work by zookeepers who were instructed to catch insects morphologically similar to triatomines. We identified triatomines using dichotomic keys (Lent & Wygodzinsky, 1979). We examined the faeces of the collected triatomines by optical microscopy according to Minuzzi‐Souza et al. (2018); fresh (400x) and Giemsa 10%‐stained (1000x) hindgut contents were examined to detect *T. cruzi* (Cuba Cuba, 1998).

We sampled blood from wild captive mammals at the Brasília Zoo between 2016 and 2017. We did not sample individuals that were difficult to contain, such as cervids, individuals debilitated due to health problems, pregnancy or breastfeeding, and old‐age animals. We collected blood after physical, chemical or physicochemical containment. The volume of blood obtained varied according to the individual's weight and their physiological and clinical characteristics. Most collections took advantage of routine containments following the Zoo's veterinarian timeline and emergency restraints, minimizing stress for the animals. Part of the collected blood (300 μl) was impregnated on filter paper, which was used for DNA extractions. We stored all collected material at −20°C.

We extracted DNA from blood samples (300 μl) according to the recommendations of the Biopur DNA Extraction Kit (Biometrix). We extracted DNA from triatomines (intestinal tissues) and sandflies (female thoraces and proximal abdominal segments) with the Illustra Tissue and Cells Genomic kit (GE Healthcare), according to Minuzzi‐Souza et al. (2018) and Machado et al. (2017), respectively. We quantified DNA samples with the NanoVue™ Plus Spectrophotometer (GE Healthcare) using the wavelength 220–330 nm; the data were expressed in ng/μL. We evaluated the purity of the samples by absorbance (A260/280), whose values should be between 1.8 and 2.0. We stored all DNA samples at −20°C.

We carried out *T*. *cruzi* quantitative PCR on the nuclear satellite DNA (nDNA‐qPCR) using primers TCZ3 and TCZ4, which amplify a fragment of 168pb. The reaction was performed in a final volume of 20 μl, with 1X of Power SYBR® Green PCR Master Mix (Applied Biosystems), 0.2 μM of each primer and 50 ng of each DNA sample. We ran qPCRs in duplicate in the StepOnePlus Real Time PCR System (Applied Biosystems) with the following conditions: 50°C for 2 min, 95°C for 10 min, 40 cycles at 95°C for 15 s, 60°C for 45 s and 72°C for 10 s (Minuzzi‐Souza et al., 2016). We analysed the results with the StepOne Software v2.3 (Applied Biosystems). Negative (mouse DNA), positive (DNA from a *T*. *cruzi* culture, according to the standard curve) and blank (sample without DNA) controls were included. A standard curve was taken for absolute quantification of the samples. The curve was based on cycle quantification values of different concentrations of *T. cruzi* DNA extracted from cultures (Berenice strain) according to Minuzzi‐Souza et al. (2018). We detected *Leishmania* in individual female sandflies by quantitative PCR of the first internal transcribed spacer of the ribosomal DNA (rDNA ITS‐qPCR) using the primers, 219 *F* (5′‐AGC TGG ATC ATT TTC CGA TG‐3′) and 219R (5′‐ATC GCG ACA CGT TAT GTG AG‐3′) (Talmi‐Frank et al., 2010) that amplify a fragment of 265‐288bp. The reaction was performed in a final volume of 20μL, with 1X of Power SYBR® Green PCR Master Mix (Applied Biosystems), 0.4 μM of each primer and 15ng of each DNA sample. qPCRs were run in 96‐well optical reaction plates (MicroAmpR), in duplicate, in the thermal cycler QuantStudio 3 Real‐Time PCR System (Applied Biosystems): 50°C for 2 min, 94°C for 12 min, 40 cycles of 95°C for 30 s, 55°C for 30 s and 72°C for 30 s. We analysed the results with the QuantStudio™ Design and Analysis Software v1.4.1 (Applied Biosystems). We included negative (mouse DNA), positive (DNA from a *L*. *infantum* culture, strain 6445, provided by the Oswaldo Cruz Institute) and blank (sample without DNA) controls. We considered samples positive when the Cycle Threshold (CT) was less or equal to 35 cycles. It is worth mentioning that during the standardization of the rDNA ITS‐qPCR it was possible to amplify samples of *L. infantum* culture with up to 1 parasite/mL.

We detected *Leishmania* in mammals using quantitative PCR targeting the conserved sequences of the *Leishmania* spp. kinetoplast minicircle DNA (kDNA‐qPCR) (Pita‐Pereira et al., 2012). The reaction mix contained 1× Power SYBR Green Master Mix (Applied Biosystems), 0.2 μM of each primer, 50 ng of template DNA and ultra‐pure distilled water to a final volume of 20 μl. We ran reactions in triplicate on 96‐well optical reaction plates (MicroAmpR) in the QuantStudio 3 Real‐Time PCR System (Applied Biosystems) based on the following conditions: initial incubation at 50°C for 2 min, 95°C for 10 min and 40 cycles of 95°C for 15 s, 53°C for 40 s, and 72°C for 8 s. We included positive (*L*. *infantum* DNA) and negative (DNA from mice) controls in all PCRs. We considered samples positive when they presented CTs less than or equal to 35 cycles. During the standardization of the kDNA‐qPCR it was possible to amplify culture samples of *L. infantum* with up to 5pg of DNA.

Our study was approved by the Ethics Committee for Animal Use (CEUA) of the Biological Sciences Institute (IB) of the University of Brasília (UnBDOC nº66711/ 2016). We captured vectors after authorization for activities of scientific purpose, Sisbio (number 33,156) issued by the Chico Mendes Institute for Biodiversity Conservation (ICMBio), and the collection of mammalian blood was authorized by Sisbio (number 54912–1) and Brasília Zoo (number 196000132/2016).

## 3 RESULTS

**Figure 2:**
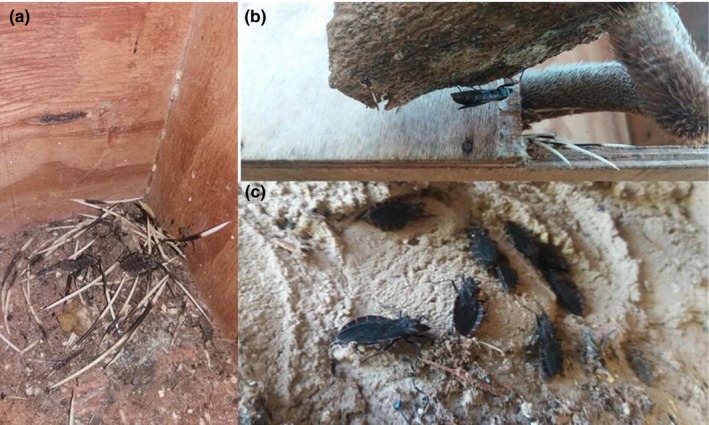
Colony of *Panstrongylus megistus* identified in the enclosure of Brazilian porcupine (*Coendou prehensilis*). (a) Inner area of the shelter with exuviaes of *P*. *megistus* nymphs. (b) *P*. *megistus* adult near the tail of the mammal. (c) *P*. *megistus* adults found behind the shelter for the Brazilian porcupine

In total, we surveyed 63 sites for triatomines between 2016 and 2017 at the Brasília Zoo and only one site was positive (1.5% infestation rate). We found a colony of *Panstrongylus megistus* in the Brazilian porcupine (*Coendou prehensilis*) unit, behind the nest box (Figure 2) on September 2016. We captured 13 females, four males, two nymphs and 32 eggs of *P*. *megistus*. Of the 16 triatomines examined, one was found infected with *T. cruzi* by microscopy (trypomastigotes and epimastigotes) and four were positive by nDNA‐qPCR. *T*. *cruzi* DNA was not detected in the porcupine blood sample.

We captured 17 phlebotomines within the following units: primates (*n* = 2), maned wolf (*n* = 2), lowland tapir (*n* = 2), crab‐eating fox (*n* = 3), puma (*n* = 4), giant anteater (*n* = 1) and Brazilian porcupine (*n* = 3). We did not capture any sandflies in the gallery forests. We identified the following species: *Evandromyia sallesi* (*n* = 3♀), *Lutzomyia longipalpis* (*n* = 4♂ and 2♀), *Nyssomyia whitmani* (*n* = 3♂ and 4♀) and *Pintomyia* sp. (*n* = 1♀). In total, we captured 13 specimens with HP‐traps and four with Shannon traps (Data S1). We did not detect *Leishmania* DNA in the sandfly samples by ITS‐qPCR.

**Figure 3:**
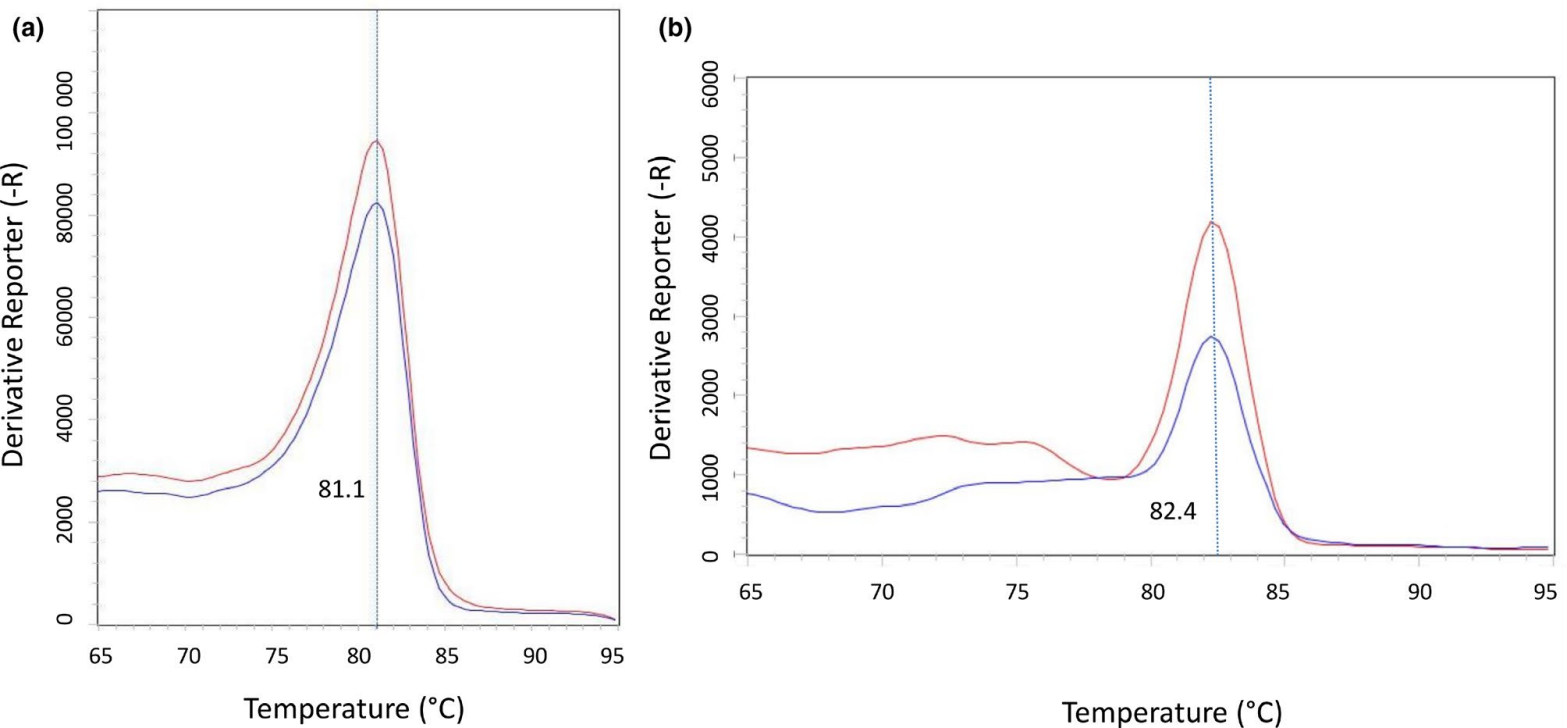
Quantitative PCR (qPCR) results. (a) qPCR of nuclear satellite DNA (nDNA‐qPCR) of *Trypanosoma cruzi*; red: ‘melt curve’ of the *T. cruzi* positive control (Berenice strain), blue: ‘melt curve’ of a mammal sample. (b) qPCR of kinetoplastid minicircle DNA (kDNA qPCR) of *Leishmania* sp.; red: ‘melt curve’ of the *L. infantum* positive control (6445 strain), blue: ‘melt curve’ of a mammal sample. Blue dashed lines represent the melt temperature of the positive samples of *T. cruzi* and *Leishmania* sp

We collected blood samples from 74 mammals, belonging to six orders, 15 families and 32 species (Table 1). nDNA‐qPCR identified 50 *T*. *cruzi‐*infected specimens (67.6%) belonging to 24 species (Figure 3). We observed highest *T*. *cruzi*‐infection rates for carnivores (89.3%) and primates (60.7%). Among primates, the highest frequency of *T*. *cruzi* was in the Golden‐headed lion tamarins (*Leontopithecus chrysomelas*), and, among carnivores, in the maned wolves (*Chrysocyon brachyurus*). Of the positive mammals, 40 individuals came from other zoological institutions or were brought by IBAMA and 10 were born at the Brasília Zoo (Table 1 and Data S1).

**Table 1:** Total mammals examined and infected by *Trypanosoma cruzi* and *Leishmania* sp. by quantitative real‐time PCR (qPCR) at the Brasília Zoo

Order/Family	Species	Specimens		
		Examined	Positive by qPCR	
			*T. cruzi*	*Leishmania* sp.
**Primates**				
Callithrichidae	*Leontopithecus chrysomelas*	6	5	1
	*Leontopithecus rosalia*	1	0	0
	*Saguinus bicolor*	2	1	0
	*Saguinus niger*	2	2	0
Aotidae	*Aotus nigriceps*	5	3^a^	1^a^
Cebidae	*Cebus albifrons*	1	1	0
Atelidae	*Alouatta caraya*	4	0	0
	*Ateles marginatus*	1	1	0
	*Lagothrix cana*	3	3	1
Pitheciidae	*Chiropotes satanas*	2	1	1
	*Callicebus cupreus*	1	0	0
**Carnivora**				
Canidae	*Cerdocyon thous*	1	1	1
	*Chrysocyon brachyurus*	7	6^c^	3
	*Lycalopex vetulus*	2	2^b^	1
	*Speothos venaticus*	1	1	0
Felidae	*Leopardus colocolo*	2	2	2
	*Leopardus pardalis*	1	0	1
	*Leopardus guttulus*	1	1	1
	*Panthera onca*	1	1	0
	*Puma concolor*	1	1	1
	*Puma yagouaroundi*	1	1	0
Mustelidae	*Lontra longicaudis*	2	2	1
Procyonidae	*Nasua nasua*	6	5^b^	3^a^
	*Procyon cancrivoros*	1	1	0
Ursidae	*Tremarctos ornatus*	1	1	0
**Pilosa**				
Myrmecophagidae	*Myrmecophaga tridactyla*	11	6^c^	4
	*Tamandua tetradactyla*	3	1	1
**Cetartiodactyla**				
Cervidae	*Odocoileus virginianus*	1	0	0
**Perissodactyla**				
Tapiridae	*Tapirus terrestris*	1	1	0
**Rodentia**				
Erethizontidae	*Coendou prehensilis*	1	0	0
Dasyproctidae	*Dasyprocta agouti*	1	0	0
Total		74	50 (67.6%, CI^d^: 61.3%–72.0%)	23 (31.1% CI:26.7%–37.3%)

a one born at Brasília Zoo

b two born at Brasília Zoo

c three born at Brasília Zoo

d 95% Confidence interval.

We detected *Leishmania* DNA in 23 individuals from 15 species (Figure 3), resulting in an infection rate of 31.1%. Infected individuals belonged to the order Carnivora (*n* = 14), Pilosa (*n* = 5) and Primates (*n* = 4); only two individuals were born at the Brasília Zoo (Table 1 and Data S1).

## 4 DISCUSSION

Our study identified triatomines and sandflies at the Brasília Zoo and revealed a high frequency of infection with *T. cruzi* and *Leishmania* spp. among captive wild mammals, mainly carnivores. Some of the mammals were born at the zoo and we suggest vector‐borne transmission. Here, we discuss ecological, epidemiological and veterinary implications of these results.

We detected the second colony of *P*. *megistus* at the Brasília Zoo. The first colony was detected in the small‐primate unit, where vector‐borne transmission of *T*. *cruzi* was confirmed (Minuzzi‐Souza et al., 2016). *Panstrongylus megistus* was also detected at the Rio de Janeiro Primatology Center (Lisboa et al., 2004), where four individuals were captured and *T*. *cruzi* transmission was proven. The presence of infected *P*. *megistus* specimens is a potential risk factor for *T*. *cruzi* transmission at the Brasília Zoo for captive mammals. However, and despite substantial sampling effort over one year, we detected only one colony, suggesting a low frequency of triatomines in the institution. Gurgel‐Gonçalves et al. (2004) showed an infection rate of 33% for *T*. *cruzi* in white‐eared opossums (*D. albiventris*) in the gallery forest area nearby the zoo but did not detect triatomines in this forest. Nevertheless, the hypothesis is that the origin of *P*. *megistus* at the Brasília Zoo would be from the adjacent gallery forests, from where they could arrive already infected considering the existence of an enzootic cycle of *T*. *cruzi*. In the FD, 894 triatomines were captured between 2012 and 2014 in 12 administrative regions, and most of them were identified as *P*. *megistus* (Minuzzi‐Souza et al., 2017)*.* In central Brazil the estimated infection rate of *P*. *megistus* is approximately 50% (Minuzzi‐Souza et al., 2018). In addition to the risk of vector‐borne transmission, the probability of oral infection should be taken into account (Shikanai‐Yasuda & Carvalho, 2012), considering that: (a) animals’ food is maintained and prepared in the zoo and could be contaminated by infected bugs, and (b) captive animals may prey on the bugs (some of these mammal species are omnivorous and may include insect in their diets) or even on infected free‐living mammals (e.g. rodents, marsupials) that inhabit the area of the institution. Vertical transmisson is also possible, but our data do not support infection from mother to offpring (see Data S1). Moreover, our previous study analysing primate samples (Minuzzi‐Souza et al., 2016) showed that three primates born to qPCR‐negative mothers at Brasília Zoo were infected with the same *T. cruzi* strain as *P. megistus* caught in their lodgings. This finding is strongly suggestive of vector‐borne transmission instead of vertical transmission, which is rare among wild primates (Lisboa et al., 2015).

We detected two important phlebotomine vector species at the Brasília Zoo: *Ny*. *whitmani* (main vector of *L*. *braziliensis*) and *Lu*. *longipalpis* (vector of *L*. *infantum*). Despite a capture effort of 7,392 hr with HP traps and 32 hr with Shannon traps, we captured a small number of sandflies (*n* = 17) when compared to other studies. Teodoro et al. (1998) captured 3,532 sandflies using Falcon traps during four months at the Maringá zoo with an effort of 408 hr. The low density of phlebotomines at Brasília Zoo may have been influenced by sampling biases such as where traps were installed, sometimes distant from animals (e.g. felines’ units), making it difficult for sandflies to access the light trap, since these insects generally fly less than 200 meters and are close to food sources. The sampling could have been complemented by other traps like Disney, oily leaves, Falcon and light traps fitted with light‐emitting diode bulbs, which could improve the efficiency of phlebotomine trapping (Lima‐Neto et al., 2018). Moreover, low phlebotomine density could be explained by seasonal variation; e.g., the abundance of *Lu. longipalpis* in Mato Grosso do Sul was higher in the wet season of the second year of sampling (Oliveira, Galati, Fernandes, Dorval, & Brazil, 2008). In addition, all methods for vector detection are imperfect; future sampling should consider other detection methods and analytical approaches (Padilla‐Torres, Ferraz, Luz, Zamora‐Perea, & Abad‐Franch, 2013;Valença‐Barbosa, Lima, Sarquis, Bezerra, & Abad‐Franch, 2014). We did not capture phlebotomines in the gallery forest adjacent to the zoo. We expected to capture sandflies in this area because they are frequent in gallery forests of the FD (Ferreira, Macedo, et al., 2015; Rapello et al., 2018). The absence of *Leishmania*‐infected sandflies may be directly related to the low number of specimens captured and the low rate of infection by trypanosomatids in the studied area (Ferreira, Minuzzi‐Souza, et al., 2015).

The rate of *T. cruzi*‐infected primates was similar that previously described by Minuzzi‐Souza et al. (2016). We found infected species that had not been studied in the previous work (*Saguinus bicolor*, *Cebus albifrons*, *Alouatta caraya*, *Ateles marginatus*, *Lagothrix cana*, *Chiropotes satanas* and *Callicebus cupreus*), whereas *Aotus nigriceps*, *L. chrysomelas* and *Saguinus niger* had already been found infected at the Brasília Zoo. To the best of our knowledge, the present study is the first to identify *T. cruzi* in *L. cana*, *A. marginatus* and *C. satanas*. We also detected canids and felids infected by *T*. *cruzi*. Zetun, Lucheis, Troncarelli, and Langoni (2014) identified *T. cruzi*‐infected crab‐eating foxes (*C. thous*) at the Sorocaba zoo, state of São Paulo. Ocelots (*L. pardalis*), maned wolves (*C. brachyurus*) and crab‐eating foxes were positive for *T. cruzi* in an area close to the Serra da Canastra National Park (Rocha et al., 2013). Albuquerque and Barreto (1968) identified *T. cruzi* in a hoary fox (*Lycalopex vetulus*) captured in the region of Franca, São Paulo State, and our study is possibly the first to confirm the infection of a bush dog (*Speothos venaticus*), a little‐known and endangered canid. Coatis (*Nasua nasua*), Neotropical otters (*Lontra longicaudis*), a crab‐eating raccoon (*Procyon cancrivorus*) and an Andean bear (*Tremarctos ornatus*) were also infected by *T. cruzi*. This parasite was isolated from coatis in the Pantanal region (Herrera et al., 2008) and from a raccoon captured in the state of São Paulo (Barretto & Ferriolli Filho, 1970). Our study showed the presence of *T*. *cruzi* in the Neotropical otter, a mustelid species that has a wide distribution in Brazil, and in *T. ornatus*, the only species of the Ursidae family in South America. In relation to the felids, it is the first report of infection in *Leopardus guttulus*, *L. colocolo* and *Puma yagouaroundi*. Among *T. cruzi*‐infected mammals born at the Brasília Zoo, two maned wolves were born to qPCR‐negative mothers, suggesting vector‐borne transmission, as already described for primates at the Brasília Zoo (Minuzzi‐Souza et al., 2016).

*Leishmania‐*infected mammals have been detected in zoos. Malta et al. (2010) identified a Black‐fronted titi monkey (*Callicebus nigrifrons*) positive for *L. infantum* at the Belo Horizonte zoo, Minas Gerais State, and Luppi et al. (2008) at this same zoo identified a *Leishmania*‐positive bush dog and a hoary fox. Six crab‐eating foxes, a bush dog, five pumas (*P. concolor*) and one jaguar (*P. onca*) were found infected by *L. infantum* at the zoo of the Federal University of Mato Grosso (Dahroug et al., 2010; Souza et al., 2010). Sastre et al. (2008) detected *L. infantum* in three gray wolves (*Canis lupus*) in Portugal and Spain.

We suggest changes in the zoo's mammal units to avoid triatomine infestation. Also, the use of deltamethrin‐impregnated collars in captive mammals should be evaluated considering their potent anti‐feeding and insecticidal effect on phlebotomines (David et al., 2001). To obtain a broader view of the trypanosomatid transmission and its impact on animal health further studies are necessary to detect *Leishmania* and *T*. *cruzi* in the other animals of the Brasília Zoo and to analyse parasite pathogenicity to the mammals. Diagnostic tests should be repeated periodically to see if new infections are occurring. With respect to the pathogenicity of *T. cruzi* and *Leishmania* in wild mammals, few data are available. Hypergammaglobulinemia, low albumin and globulin levels, and cardiac alterations were observed in *T*. *cruzi*‐infected golden lion tamarins (Monteiro et al., 2006; Monteiro, Dietz, & Jansen, 2010). After an 11‐year follow‐up research with *L. rosalia* and *L*. *chrysomelas*, Lisboa et al. (2015) concluded that these species maintain the infection for a long period and are excellent reservoirs for *T*. *cruzi*. There is evidence of *Leishmania*‐infected mammals with clinical signs. A *Leishmania infantum*‐infected black‐fronted titi monkey (*Callicebus nigrifrons*) presented clinical signs of Visceral Leishmaniasis and died at the Belo Horizonte zoo (Malta et al., 2010); a bush dog and a hoary fox had also symptomatic Visceral Leishmaniasis in this zoo (Luppi et al., 2008). We recommend that veterinarians working with such species pay attention to signals of leishmaniasis and Chagas disease. Future studies should reveal the real impact of *Leishmania* spp. and *T. cruzi* infection in wild mammals.

## 5 CONCLUSIONS

We detected *T*. *cruzi* in five groups of mammals (Carnivora, Cetartiodactyla, Perissodactyla, Pilosa and Primates) at the Brasília Zoo. Our results suggest vector‐borne transmission of *T. cruzi* among maned wolves; measures to reduce the risk of new infections should therefore be taken. We also report sandfly presence and *Leishmania*‐infected mammals at the Brasília Zoo. Translocation of wild mammals in and out of the Brasília Zoo should consider the risk of *T. cruzi* and *Leishmania* spread.

## 6 ACKNOWLEDGEMENTS

We are grateful to the technical staff of the Brasília Zoo, including zookeepers, veterinarians and biologists. We also thank Mariana Gallego Bessa, Marcos Takashi Obara, Elisa Neves Vianna and Ludmilla Aguiar for reviewing the manuscript. This research was partially supported by the Fundação de Apoio à Pesquisa do Distrito Federal‐FAP DF, Universidade de Brasília and Conselho Nacional de Desenvolvimento Científico e Tecnológico ‐ CNPq.

## CONFLICT OF INTEREST

None.

## ETHICAL STATEMENT

The authors confirm that the ethical policies of the journal, as noted on the journal's author guidelines page, have been adhered to and the appropriate ethical review committee approval has been received. The US National Research Council's guidelines for the Care and Use of Laboratory Animals were followed.
