# Supplementary material for: Trypanosomatid infections in captive wild mammals and potential vectors at the Brasilia Zoo, Federal District, Brazil
Source: Vet Med Sci. 2019 Nov 19;6(2):248–56. doi: 10.1002/vms3.216 (PMC7196675; doi:10.1002/vms3.216)
Supplement: Supplementary file 1 [file VMS3-6-248-s001.docx]

Supplementary data. List of mammals infected by *Trypanosoma cruzi* and *Leishmania* sp., characteristics and quantitative real-time PCR (qPCR) results at the Brasília Zoo.

|  |  |  | qPCR result | |
| --- | --- | --- | --- | --- |
| Species | Origin | Month and year of birth or arrival at Zoo | *T. cruzi* | *Leishmania* |
| *Chrysocyon brachyurus* | Born at Brasília Zoo^a^ | July 2013 | + | - |
| *Chrysocyon brachyurus* | Born at Brasília Zoo | July 2013 | + | - |
| *Chrysocyon brachyurus* | Born at Brasília Zoo | July 2015 | + | - |
| *Chrysocyon brachyurus* | Wild born - IBAMA – DF | No data | + | + |
| *Chrysocyon brachyurus* | Wild born - IBAMA – DF | March 2012 | + | - |
| *Tremarctos ornatus* | Born at Sapucaia do Sul Zoo – RS | September 2017 | + | - |
| *Procyon cancrivoros* | Wild born - IBAMA – DF | July 2014 | + | - |
| *Puma concolor* | Wild born - IBAMA – GO | October 2013 | + | - |
| *Lontra longicaudis* | Wild born - IBAMA – DF | September 2015 | + | - |
| *Speothos venaticus* | Wild born - IBAMA – PA | August 2017 | + | - |
| *Leopardus colocolo* | Wild born - IBAMA | June 2009 | + | + |
| *Leopardus colocolo* | Wild born - IBAMA – DF | November 2014 | + | + |
| *Nasua nasua* | Born at Brasília Zoo | September 2009 | + | + |
| *Leopardus tigrinus* | Wild born - IBAMA – DF | July 2014 | + | + |
| *Nasua nasua* | Wild born - IBAMA – DF | September 2008 | + | + |
| *Lycalopex vetulus* | Wild born - IBAMA – DF | November 2009 | + | + |
| *Nasua nasua* | Wild born - IBAMA – DF | September 2008 | + | - |
| *Panthera onca* | Born at Zoo Salvador – BA | July 2012 | + | - |
| *Cerdocyon thous* | Wild born - IBAMA – DF | June 2014 | + | + |
| *Nasua nasua* | Wild Born - IBAMA – DF | September 2008 | + | + |
| *Nasua nasua* | Born at Brasília Zoo | September 2009 | + | - |
| *Lycalopex vetulus* | Born at Brasília Zoo | August 2009 | + | - |
| *Chrysocyon brachyurus* | Wild born - IBAMA – DF | No data | + | + |
| *Puma yagouaroundi* | Born at Bauru Zoo – SP | November 2007 | + | + |
| *Lontra longicaudis* | Wild born - IBAMA – DF | No data | + | + |
| *Tapirus terrestris* | Wild born - IBAMA – DF | January 2016 | + | - |
| *Tamandua tetradactyla* | Wild born - IBAMA – DF | December 2012 | + | + |
| *Myrmecophaga tridactyla* | Born at Brasília Zoo | January 2006 | + | - |
| *Leontopithecus chrysomelas* | Born at São Paulo Zoo – SP | January 2013 | + | - |
| *Myrmecophaga tridactyla* | Born at Brasilia Zoo | July 2011 | + | - |
| *Myrmecophaga tridactyla* | Wild born - IBAMA – DF | April 2010 | + | - |
| *Myrmecophaga tridactyla* | Born at Goiania Zoo – GO | September 2001 | + | + |
| *Myrmecophaga tridactyla* | Born at Brasília Zoo | September 2010 | + | - |
| *Myrmecophaga tridactyla* | Wild born - IBAMA – AM | May 2017 | + | + |
| *Leontopithecus chrysomelas* | Born at São Paulo Zoo – SP | November 2013 | + | - |
| *Leontopithecus chrysomelas* | Born at São Paulo Zoo – SP | November 2013 | + | - |
| *Leontopithecus chrysomelas* | Born at São Paulo Zoo – SP | November 2013 | + | - |
| *Leontopithecus chrysomelas* | Born at São Paulo Zoo – SP | November 2013 | + | + |
| *Saguinus niger* | Born at Ararajuba Ipê – MA | October 2007 | + | - |
| *Aotus nigriceps* | Born at Brasília Zoo | January 2012 | + | - |
| *Cebus albifrons* | Wild born - IBAMA – AC | July 2006 | + | - |
| *Lagothrix cana* | Born at Ararajuba Ipê – MA | July 2006 | + | + |
| *Ateles marginatus* | Wild born - IBAMA | June 1996 | + | - |
| *Saguinus niger* | Wild born - IBAMA – PA | September 2008 | + | - |
| *Aotus nigriceps* | Wild born - IBAMA - AC | September 2006 | + | - |
| *Aotus nigriceps* | Wild born - IBAMA – DF | October 2007 | + | - |
| *Lagothrix cana* | Wild born - IBAMA – AM | May 2017 | + | - |
| *Lagothrix cana* | Wild born - IBAMA – AM | May 2017 | + | - |
| *Saguinus bicolor* | Wild born - IBAMA – AM | August 2017 | + | - |
| *Chiropotes satanas* | Wild born - IBAMA – PA | August 2006 | + | + |
| *Aotus nigriceps* | Born at Brasília Zoo | November 2010 | - | + |
| *Chrysocyon brachyurus* | Wild born - IBAMA – DF | April 2009 | - | + |
| *Leopardus pardalis* | Wild born - IBAMA – AM | May 2017 | - | + |
| *Myrmecophaga tridactyla* | Wild born - IBAMA – DF | No data | - | + |
| *Myrmecophaga tridactyla* | Wild born - IBAMA – MS | December 2000 | - | + |

IBAMA Instituto Brasileiro do Meio Ambiente e Recursos Renováveis. Brazilian states: AC Acre, AM Amazonas, BA Bahia, DF Distrito Federal, GO Goiás, MA Maranhão, MG Minas Gerais, PA Pará, SP São Paulo

Supplementary data. Number of sandflies captured at the Brasília Zoo: location, sex, type of trap (HP or Shannon), species, year, month of sampling.

| Location (mammals’ unit)^a^ | Female | Male | HP | Shannon | Species | Month/Year |
| --- | --- | --- | --- | --- | --- | --- |
| P12 (primates) | 1 | 0 | 1 | 0 | *Evandromyia sallesi* | September 2016 |
| P13 (primates) | 0 | 1 | 1 | 0 | *Nyssomyia whitmani* | September 2016 |
| P14 (maned wolf) | 1 | 1 | 2 | 0 | *Nyssomyia whitmani* | September 2016 |
| P15 (lowland tapir) | 1 | 0 | 1 | 0 | *Nyssomyia whitmani* | September 2016 |
| P16(crab-eating fox) | 1 | 0 | 1 | 0 | *Nyssomyia whitmani* | November 2016 |
| P18 (giant anteater) | 1 | 0 | 1 | 0 | *Lutzomyia longipalpis* | November 2016 |
| P15 (lowland tapir) | 1 | 0 | 1 | 0 | *Evandromyia sallesi* | March 2017 |
| P20 (puma) | 0 | 4 | 4 | 0 | *Lutzomyia longipalpis* | March 2017 |
| P21 (Brazilian porcupine) | 1 | 0 | 1 | 0 | *Evandromyia sallesi* | May 2017 |
| P21 (Brazilian porcupine) | 0 | 1 | 0 | 1 | *Nyssomyia whitmani* | June 2017 |
| P16 (crab-eating fox) | 1 | 0 | 0 | 1 | *Nyssomyia whitmani* | June 2017 |
| P16 (crab-eating fox) | 1 | 0 | 0 | 1 | *Lutzomyia longipalpis* | June 2017 |
| P21 (Brazilian porcupine) | 1 | 0 | 0 | 1 | *Pintomyia sp.* | June 2017 |
|  |  |  |  |  |  |  |

^a^ Figure 1 shows the distribution of HP-traps at the Brasília Zoo.
